# A case of myocarditis in a 60-year-old man 48 h after mRNA vaccination against SARS-CoV2

**DOI:** 10.1007/s00392-021-01946-4

**Published:** 2021-11-03

**Authors:** Dirk Habedank, Antonia Lagast, Monica Novoa-Usme, Iskandar Atmowihardjo

**Affiliations:** 1 DRK Kliniken Berlin Köpenick, Medizinische Klinik Kardiologie, S.-Allende-Str. 2-8, 12555 Berlin, Germany; 2 Universitätsmedizin Greifswald, Klinik und Poliklinik für Innere Medizin B, Fleischmannstr. 8, 17475 Greifswald, Germany

**Keywords:** mRNA, Vaccination, mRNA-1273, SARS-CoV2, Myocarditis, MRI, Troponin, 60 years old

Sirs: 

According to the recent analysis of the US Center for Disease Control (CDC), a number of 56–69 cases of myocarditis per 1 million mRNA-vaccine doses have to be expected in the age group of 12–17 years in males and of only 3–4 cases in the age group $\ge$ 30 years [1], the latter number also corresponding to the latest report from August 2021 of the responsible German authority (Federal Institute for Vaccines and Biomedicines, Paul-Ehrlich-Institut) [2]. The estimated number of deaths prevented by COVID-19 vaccine in the age group $\ge$ 30 years is 700, thus proving the overwhelming net benefit of the vaccination. The US vaccine safety passive monitoring system reported as to July 22, 2021* n* = 497 patients meeting the CDC’s case definition of myocarditis, but the median age was 19 years only [3].

Against this background, we report on a 60-year-old man with normal body mass index (189 cm, 84 kg) and empty cardiovascular anamnesis despite a WHO degree I arterial hypertension. On July 1st, 2021, he received his 2nd dose of mRNA-vaccine (mRNA-1273, Moderna), and developed a typical vaccination reaction with 39 °C fever and dizziness. About 24 h after vaccination, he had palpitations and after 36 h, he fainted and lost consciousness for some seconds. He was admitted to our hospital where the ECG showed sinus rhythm and no signs of ischemia. Fast track echocardiography was normal, but the troponin T (Roche Cardiac T cobas h232) at admission was elevated to 160 ng/L (cutoff 50 ng/L) and together with the preceding syncope an urgent coronary angiography was indicated. A coronary heart disease was excluded this way. A cardiac magnet resonance imaging (CMRI) was performed next day (80 h after vaccination) and showed a focal edema of anterolateral medial wall in the T2 sequences (Fig. 1a) and a corresponding subepicardial late enhancement in the short axis and four-chamber view (Fig. 1b, c), both consistent with the diagnosis of a myocarditis. The patient remained free of symptoms, the troponin declined to the normal range and he was discharged at day 5. The echocardiography a week and a month later remained normal, so that we presume a complete recovery.

**Fig. 1:**
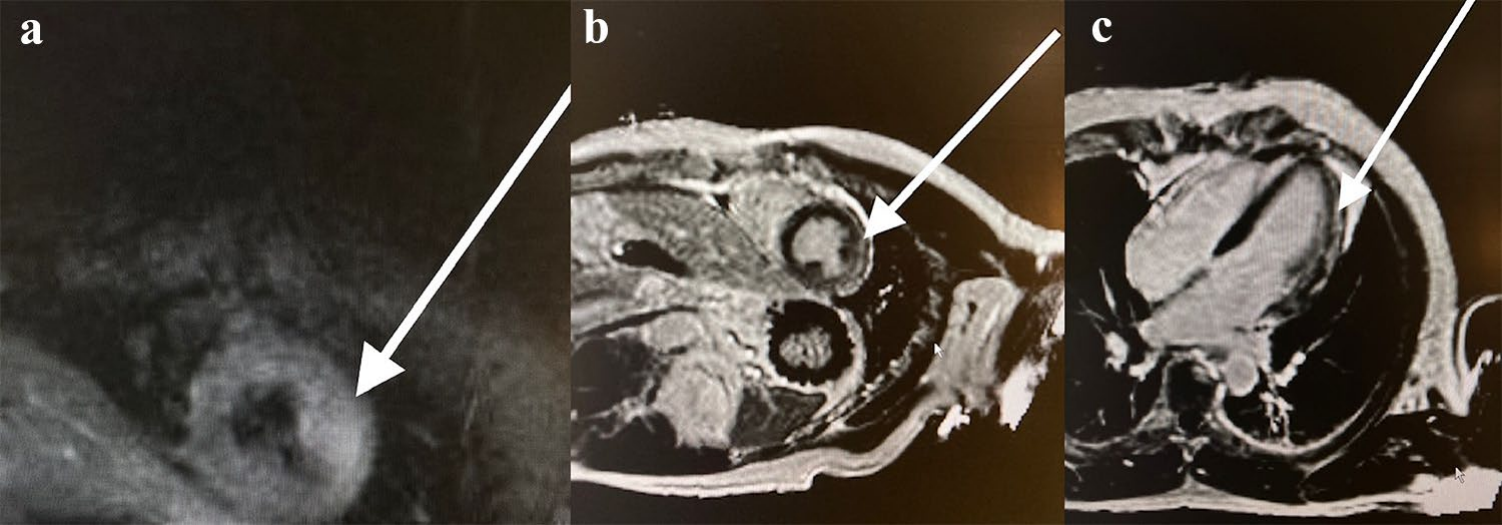
**a** Cardiac MRI, T2 sequence, arrow indicating focal edema anterolateral medial (criterion 1 in ESC definition). ** b** Short-axis view showing subepicardial gadolinium late enhancement anterolateral medial (criterion 3 in ESC definition [4]).** c** Corresponding four-chamber view

Our patient presented a typical pattern of transient clinical symptoms and MRI images of myocarditis in a very close and suggestive time relation; however, a causality between the mRNA vaccination and the myocarditis has not been proven. The CDC case definition of confirmed myocarditis [1] is fully met by (a) palpitations and syncope ($\ge$ 1 clinical symptoms), (b) CMRI findings consistent with myocarditis [4], (c) troponin level above upper limit and (d) exclusion of other causes. In contrast to the increasing number of reported cases of myocarditis after mRNA vaccination, our patient was 60 years old, and in this age group, no case of vaccine-related myocarditis has been reported to the responsible German authority up to now [2]. We classified the patient in a low-risk group because of his rapid and complete clinical recovery, the return of troponin into normal values within 24 h and the normal LVEF in both echocardiography and CMRI. We, therefore, refrained from both endomyocardial biopsy and wearable defibrillator vest. Until today, there are no cases with proven ventricular arrhythmias due to vaccine-induced myocarditis. The only published case with cardiac arrest associated with mRNA1237 was caused by pericardial tamponade [5]. A similar benign course of myocarditis has been reported in a large cohort within the U.S. military health system, summing up to 23 cases per 2.9 million vaccine doses [6]. Another series of 6 case reports from Israel included 5 patients between 16 and 29 years, which did not need a coronary angiography because of the younger age [7]. The majority of cases occurred within 6 days after 2nd vaccination [2]. Both the mRNA-1273 (SpikeVax, Moderna) and the BNT162b2 (Comirnaty, BioNTech-Pfizer) vaccine could induce a myocarditis [8, 9], caused perhaps by a hypothesized molecular mimicry between the spike protein of SARS-CoV2 and self-antigens [10]. Furthermore, “dysregulated immune response to mRNA, activation of immunological pathways, and dysregulated cytokine expression” have been proposed as mechanisms [10]. To our knowledge, nearly all reported patients recovered completely and one must keep in mind that there is a significantly higher risk of myocarditis from COVID-19 infection itself compared to COVID-19 vaccination [10–12]. The latest report of the German Paul-Ehrlich-Institut from August 19, 2021 listed 393 cases of peri- or myocarditis per 69 million doses of BNT165b2 (BioNTech-Pfizer) and 49 cases per 8.5 million doses of mRNA-1273 (Moderna) [2]. This gave reason to an updated product information and a red-hand-letter of the responsible authority which indicate explicitly on this complication. Our short report also intends to alert physicians and authorities to this rare side effect of mRNA vaccination.

## Abbreviations

BNT165b2: BioNTech-Pfizer COVID-19 vaccine
CDC: Center for Disease Control of the USA
CMRI: Cardiac magnetic resonance imaging
mRNA-1273: Moderna COVID-19 vaccine
PEI: Paul-Ehrlich-Institut
WHO: World Health Organization
